# Prostate-Specific Antigen Doubling Time Kinetics following Radical Prostatectomy to Guide Need for Treatment Intervention: Validation of Low-Risk Recurrences

**DOI:** 10.3390/cancers14174087

**Published:** 2022-08-24

**Authors:** Erica Huang, Joshua Tran, Linda My Huynh, Douglas Skarecky, Robert H. Wilson, Thomas Ahlering

**Affiliations:** 1 Department of Urology, University of California, Irvine, CA 92868, USA; 2 Scholars Program, University of Nebraska Medical Center, Omaha, NE 68198, USA; 3 Health Policy Research Institute, University of California, Irvine, CA 92697, USA

**Keywords:** prostate cancer, surgical therapy, decision analysis, biochemical recurrence, PSA

## Abstract

**Simple Summary:**

Biochemical recurrence following radical prostatectomy is concerning but does not accurately predict cancer progression or death. In our patients with a biochemical recurrence, we explore whether PSA doubling time kinetics can safely guide avoidance of treatment, preventing unwanted side effects and costs. In our study, initial PSADT and subsequent DT patterns were the only predictors of no need for treatment. Gleason grade group, pathological stage, preoperative PSA, and age were not significant predictors of treatment. Approximately one-third of BCRs observed in this cohort following RP were determined to be low-risk and able to be safely followed without treatment using PSADT kinetics, with a 100% cancer-specific survival after 7.6 years of follow-up.

**Abstract:**

Biochemical recurrence (BCR) following radical prostatectomy (RP) has a limited ability to predict prostate cancer (PC) progression, leading to overtreatment, decreased quality of life, and additional expenses. Previously, we established that one-third of men with BCR in our group experienced low-risk recurrences that were safely observed without treatment. Our retrospective cohort analysis of 407 BCR patients post RP validates the use of PSA doubling time (DT) kinetics to direct active observation (AO) versus treatment following RP. The primary outcome was no need for treatment according to the predictive value of models of ROC analysis. The secondary outcome was PC-specific mortality (PCSM) according to Kaplan–Meier analysis. A total of 1864 men underwent RP (June 2002–September 2019); 407 experienced BCR (PSA > 0.2 ng/dL, ×2), with a median follow-up of 7.6 years. In adjusted regression analysis, initial PSADT > 12 months and increasing DT were significant predictors for AO (*p* < 0.001). This model (initial PSADT and DT change) was an excellent predictor of AO in ROC analysis (AUC = 0.83). No patients with initial PSADT > 12 months and increasing DT experienced PCSM. In conclusion, the combination of PSADT > 12 months and increasing DT was an excellent predictor of AO. This is the first demonstration that one-third of BCRs are at low risk of PCSM and can be managed without treatment via DT kinetics.

## 1. Introduction

Prostate cancer (PC) recurrence following definitive therapy is common, with prevalence ranging from 20 to 40% [1]. Following radical prostatectomy (RP), prostate-specific antigen doubling time (PSADT) has been shown to be a predictor of prostate cancer (PC) progression, metastases, and PC-specific mortality (PCSM) [2]. Whereas the American Urological Association recommends the use of PSA kinetics primarily to distinguish between local and distant recurrence, no other groups have explored the use of PSADT in post-recurrence active observation (AO) [3]. A 2019 study by Matsumoto et al. revealed that observation of patients with recurrent PC was a viable option in patients with Gleason ≤ 7 disease and PSADT ≥ 6 months; however, the authors did not propose a process to safely observe patients [4]. Similarly, the European Association of Urology (EAU) makes a “weak” recommendation of watchful waiting for patients with PSADT > 12 months [5]. 

In 2005, our group published long-term PSA outcomes of 207 men following RP (between 1984 and 1994) at the Long Beach VA medical center. Treatment was managed under the guidance and authority of the local VA Medical Oncology Tumor Board; a total of 90 (44%) patients developed PSA recurrence. Throughout the study period, androgen deprivation therapy (ADT) was administered continuously (usually via surgical castration or estrogen therapy). Average follow-up for the study was ten years. In group 1 (*n* = 15), all 15 patients died of PC following failure of ADT at an average of 7.6 years (range: 2.9–14.5 years). In group 2 (*n* = 39), all patients received continuous ADT, and 10 (26%) died of non-PC causes. In group 3 (*n* = 36), in which none of the patients had undergone treatment, 14 (40%) died of non-PC causes. The average PSADT in groups 1–3 was 6, 11, and 30 months, respectively. Analysis revealed that clinical factors impacting PCSM, including age, pre operative PSA, Gleason grade 4–5, seminal vesicle involvement, lymph node positivity, time to PSA elevation and PSADT only PSADT, and Gleason grade 4–5 disease, were statistically significant [2].

In the present study of 407 men with post-RP BCR, three groups were stratified by PSADT. First, approximately one-third of men with BCR experienced immediate rapid progression undergoing adjuvant/early treatment intervention without establishing PSADT. Second, one-third had a rising PSA, although not as early or aggressively as the first group, but still requiring treatment with salvage ADT and/or RT. The final one-third had gradually rising PSA and PSADT and were managed with active observation based on PSA kinetics without ADT and/or RT with no PC mortality [6]. With this study, we seek to validate the use of both “initial” PSADT and subsequent changes in the PSADT pattern over time to identify men who can be observed safely without intervention versus those requiring ADT and/or RT intervention.

## 2. Methods

### 2.1. Patients

A retrospective review of prospectively collected data was conducted for patients undergoing robot-assisted RP by a single surgeon between June 2002 and September 2019. Preoperative demographics, oncologic information, and long-term follow-up were prospectively recorded in an anonymized, electronic database under approved institutional review board protocol at the University of California, Irvine (HS#1998–1984). The database was frozen for statistical analysis based on follow-up through 29 March 2021. All data were collected in compliance with the Health Insurance Portability and Accountability Act (HIPAA), and federal guidelines for informed consent were followed. 

Active PSA observation began at serial elevation of the PSA > 0.1 ng/mL. Biochemical recurrence was defined as two consecutive PSA values greater than or equal to 0.2 ng/mL or adjuvant therapy prior to first PSA value after RP. Patients were counseled about treatment interventions, such as RT and/or ADT, when PSA > 0.1 ng/mL and observed according to European Association of Urology (EAU) guidelines. Treatment interventions were guided by previous studies indicating that patients with initial PSADT < 12 months and high pathological GGG and stage are at higher risk of cancer progression [3,4,5,7]. In a similar fashion, patients classified as EAU low-risk (PSADT > 12 months, pGS < 8) were counseled about the option of observation [5].

After excluding patients undergoing cytoreductive (*n* = 3) or simple prostatectomy (*n* = 9), patients with neuroendocrine/small cell adenocarcinoma (*n* = 3), and those who underwent chemotherapy indicating more aggressive disease and metastases (*n* = 4), 1865 patients were identified, of which 407 patients experienced BCR (*n* = 364) or underwent adjuvant intervention due to advanced pathologic grade and/or disease stage (*n* = 43). All patients in this group were followed-up over 6 months (May 2020–October 2020) via phone call (×3), email, patient appointment, and/or mail to ensure that the most recent and up-to-date information was included. Of these 407 patients who experienced BCR, 271 patients were included in the treatment group (RT and/or ADT), whereas 136 patients did not undergo any secondary treatment (ADT and/or RT) and remained in the active observation group.

PSADT graphs of the 407 patients include post-RP PSA values and dates. PSADT was calculated using a natural log growth function, with all post-BCR PSA values (natural log of 2 (0.693) divided by the slope of the relationship between the log of PSA and time of PSA measurement for each patient) [8]. The “initial” PSADT was calculated with the first four PSA values after BCR. Patients (*n* = 162) without initial PSADT and DT pattern in the treatment group did not have enough PSA values prior to treatment due to rapid PSA progression. In the remaining patients (*n* = 245), PSA progression provided an adequate number of PSA values to establish (1) an “initial” PSADT and (2) increasing or decreasing patterns. To be considered in the AO group, PSADT pattern was needed for 30+ months postoperatively based on continuous calculation. Those without a measurable DT pattern in this group did not have the four required PSA values prior to non-cancer specific death (*n* = 2), lost to follow-up (*n* = 1), and after BCR (*n* = 1). In the treatment group, PSADT pattern was identified based on PSA progression prior to treatment intervention. 

### 2.2. Statistical Methods and Analysis

Student’s *t*-tests were conducted for continuous variables, and test of proportions or ANOVA were performed for categorical variables to evaluate demographic and oncologic differences between the observation and treatment groups. Significance is defined as *p* < 0.05. Fifteen-year overall and PC-specific survival was evaluated with Kaplan–Meier analysis and stratified between the observation and treatment groups. Patients were censored at the date of death or last known follow-up.

Univariate and multivariate logistic regression models were utilized to evaluate predictors of treatment. Possible predictors were determined a priori and included initial PSADT and DT change as the primary exposure variables and preoperative PSA, pGGG, age, and p-stage as secondary variables. Variables were selected based on univariate regression models, literature, and expert opinion (TA) [3,4,7,9,10,11]. Preoperative PSA and age were measured as continuous variables, and PSADT change (increasing or decreasing DT), pGGG (grade groups 1–3 or 4–5), p-stage (pT2 or pT3/pT4), and PSADT (<12 months or >12 months) were measured as categorical variables. A backward logistic regression model was applied to obtain the final multivariate model, including both categorical initial PSADT and DT change. Receiver–operator characteristic (ROC) curve analysis was conducted to evaluate the predictive value of each model. Ad hoc regression analysis was also performed, with PCSM as the outcome variable and the same secondary variables. All statistical tests were conducted and all figures were produced with R statistical package (R Foundation for Statistical Computing, Vienna, Austria).

## 3. Results

**Figure 1:**
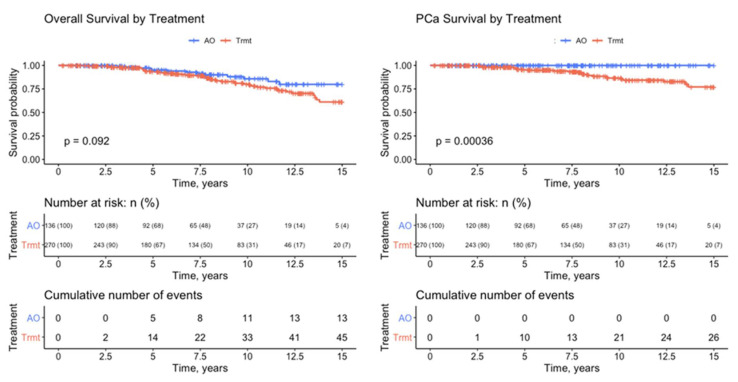
Kaplan–Meier analysis of overall survival and prostate-cancer-specific survival in the active observation (*n* = 136) and treatment (*n* = 271) groups.

Table 1 illustrates the demographics of patients in the active observation versus treatment arms. Patients in each group were of similar age, with similar mean (SD) follow-up periods of 7.5 (4.0) and 7.7 (4.4) years following RARP, respectively. Patients in the treatment group had higher risk features in terms of preoperative PSA, positive margins, p-stage, and pGGG. The proportion of patients with an increasing PSADT pattern was significantly higher in the active observation group (*p* < 0.001) than in the treatment group. Overall survival and prostate-cancer-specific survival (PCSS) were significantly higher in the observation group (*p* < 0.001) than the treatment group. Furthermore, 15-year Kaplan–Meier survival curves show a significant difference in PCSS between the active observation and treatment groups in 15-year analysis (*p* < 0.001), and the difference in overall survival was trending (0.092). At 15 years, the PCSS was 100% vs. 77% in the active observation group vs. treatment group (Figure 1). Eight-year Kaplan–Meier analysis, closer to the median 7.6 years follow-up, similarly demonstrated 100% vs. 92% PCSS (*p* = 0.0057) and 90% vs. 86% overall survival (*p* = 0.35) in active observation vs. treatment group.

Table 2A,B illustrates univariate and multivariate models of predictors of active observation. In univariate analysis, initial PSADT and PSADT patterns were the only significant indicators of no treatment. GGG, preoperative PSA, p-stage, and age were not significant predictors of no treatment. In multivariate analysis, only initial PSADT and DT change remained significant in the model. Patients with initial PSADT > 12 months had an 8.9 times increased likelihood of not needing treatment compared to those with PSADT < 12 months, and those with increasing DT patterns had a 5.5 times increased likelihood of not needing treatment compared to those with decreasing DT patterns. ROC analysis yielded an AUC of 0.835.

Table 2C illustrates the multivariate model of initial PSADT. In univariate analysis, there were no significant predictors of PCSM. However, backward regression analysis showed only initial PSADT to be a significant predictor of no need for treatment (OR: 8.74, 95% CI: 5–15.28, *p* < 0.001, AUC: 0.80). Similarly, to examine the strength of the predictive models if only DT change was included, the same multivariate backward regression analysis was conducted with DT change, GGG, preoperative PSA, p-stage, and age (Table 2D). DT pattern was the only remaining significant predictor of no treatment (OR: 5, 95% CI: 2.95–8.48, *p* < 0.001, AUC: 0.7458). 

In ad hoc regression analysis for PCSS, PSADT pattern and age were significant predictors of PCSS. Patients with an increasing DT pattern had an approximately 9.7 times increased likelihood of PCSS (*p* = 0.006) relative to those with a decreasing DT pattern (Supplementary Table S1). ROC analysis yielded an AUC of 0.78. 

## 4. Discussion

This is one of the first original studies to report the existence of very low-risk prostate cancer recurrences following RP that do not require secondary treatment. Conceptually, it appears that cells escape the prostate prior to surgery and typically land in the pelvis (i.e., lymph nodes and nerves), causing an eventual PSA recurrence. Unlike other cancers, a subset of cells seemingly lose the ability to further metastasize and progress. Whereas recurrent disease is typically treated universally, the results of the present study suggest the existence of three types of prostate cancer recurrence: an adjuvant treatment group, a salvage group, and a low-risk group that does not necessitate intervention.

In this study of 407 patients with post-RP BCR, the “initial” PSADT was calculated from the four initial PSA values at and above 0.2 ng/mL. Following the initial PSADT, the PSADT pattern was (re)calculated with each ensuing PSA value and date, and both the initial and pattern were significant, independent predictors of successful active observation. These observations are the first to validate the use of PSADT in guiding active observation in at least one-third of patients with BCR. In 15-year Kaplan–Meier analysis, no active observation patients experienced PCSM, compared to 23% in the treatment group (Figure 1). 

**Figure 2:**
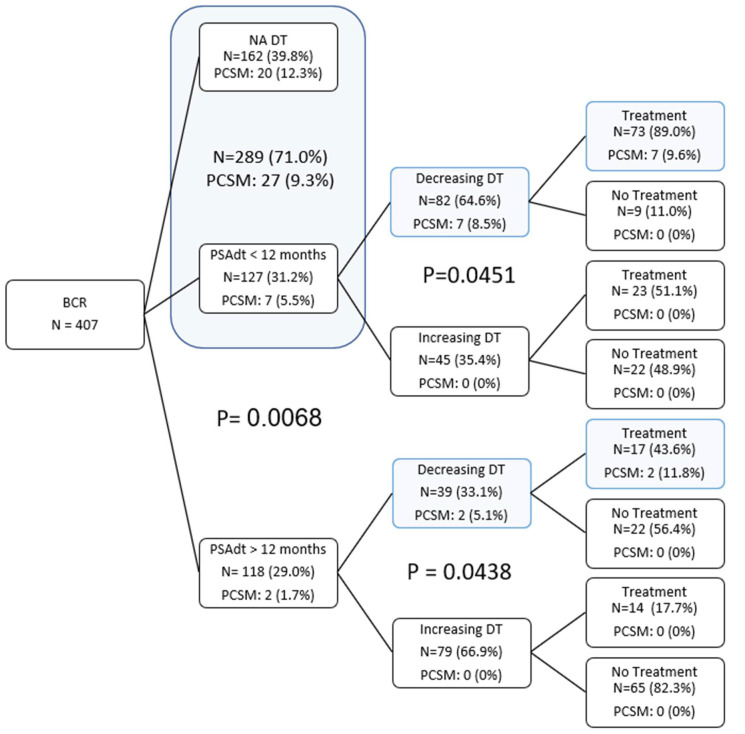
Tree diagram demonstrating PCSM by first initial PSADT after BCR, with the subsequent DT pattern.

Systematic identification of patients in the low-risk active observation group is crucial for minimizing overtreatment of BCR. Whereas oncologic characteristics such as preoperative PSA, surgical margin status, p-stage, and pGGG have been traditionally used [5], the present study shows both initial PSADT and PSADT pattern to be stronger, independent predictors of need for treatment. Furthermore, of the patients in the active observation group, approximately 20% were traditionally high-risk for pGGG 4–5 disease, 50% had pT3/T4 disease, and 27% had positive surgical margins. Despite a high proportion of these adverse features, initial PSADT was >12 months in 79.4% of patients, and the PSADT pattern was increasing in 72% of patients (Figure 2). These findings emphasize the importance of longitudinal PSA observation and are consistent with the current paradigm shift in PC management of basing treatment on pathologic characteristics. Van den Broek et al. recently suggested that short PSADT and high biopsy Gleason scores are most consistent with adverse outcomes post RP and that those with longer PSADT and lower Gleason scores are at lower risk [12]. Thus, the salvage therapy approach has replaced adjuvant radiotherapy [13,14].

Central to the success of active observation is stepwise risk stratification using initial PSADT, in addition to a continuous PSADT pattern. Whereas the AUC of initial PSADT and PSADT patterns was 0.80 and 0.75, respectively, their combined predictive capability yielded a significantly increased AUC of 0.835. In practice, when men were assessed utilizing PSADT kinetics following BCR, 127/244 (52%) were eligible for active observation after considering both initial PSADT and PSADT pattern. However, if only the initial PSADT was used, 159/244 (65%) would be eligible, yielding 32 (13%) patients who would be overtreated. Whereas the model with initial PSADT is highly sensitive (0.77) and specific (0.73) itself, DT pattern is more sensitive (0.82), and its inclusion in the model improves the ability to predict those who truly do not need treatment. These findings emphasize the need for continued and longitudinal observation of patients with BCR with PSADT pattern. 

Given the continued and longitudinal use of the PSADT pattern, the presently proposed method of BCR risk stratification is novel and exceptional. Current methods for determining risk of cancer progression are heterogenous and rely almost exclusively on surgical pathology. In 2011, Eggener and colleagues developed and evaluated a nomogram-predicting 15-year PCSM, including pGGG 4-5, seminal vesicle invasion (SVI), and surgery year, with an externally validated concordance index of 0.92 [15]. Similarly, a 2014 study by Abdollah et al. evaluated 10-year PCSS. However, this nomogram was restricted to only patients with node-positive cancer (pN1) [16]. This model yielded a predictive capability ranging from 0.795 to 0.833, although external validation by Bianchi et al. later found it to overpredict PCSS, with an AUC of 0.658 [17]. In 2015, Brockman et al. published the first nomogram to utilize post-RP characteristics to predict PCSM, incorporating preoperative PSA, pathological Gleason grade group (pGGG), extraprostatic extension, SVI, time to BCR, PSA at time of BCR, and PSADT to predict PCSM. This model was internally validated to yield an AUC of 0.774 for 10-year survival [7]. When compared to the models used in the present study, an AUC of 0.835 not only represents a significant improvement overall but also enables a significant reduction in overtreatment. Most recently, a systematic review of current literature to identify clinical and tumor features with an independent prognostic impact on oncologic outcomes was conducted by Van den Broek et al. This review similarly established that PSADT had the most pronounced association with oncologic outcomes after RP, with shorter PSADT associated with an increased risk of developing adverse oncological outcomes. [12] Thus, it should follow that if any individual’s PSADT changes throughout disease course, their oncologic risk should as well.

In this paper, we introduce a new paradigm whereby treatment decisions only occur after PSA values are established rather than at the time of pathological review. Our novel treatment algorithm utilizes the initial PSADT and PSADT pattern, demonstrating that pathologic characteristics, such as grade and stage, are no longer metrics associated with need for treatment or PCSM. Ad hoc analysis of PCSS showed the PSADT pattern to be a significant and independent predictor of PCSS (OR = 7.18, *p* = 0.028), whereas initial PSADT was not found to be a significant predictor, highlighting the importance of longitudinal follow-up and continuous risk stratification. As the PSADT pattern can continuously change, it is intuitive that it is a more durable indicator of long-term survival outcomes. 

The present results should be considered within the context of study design. PCSS was not the primary outcome due to the small number of overall mortality events. As such, multivariate analysis was conducted, with need for treatment as a surrogate for identifying patients at-risk for PCSM. However, to compare the mortality rates reported in the present study with nomogram risks published in literature, risk of PCSM at 5, 10, and 15 years based on the nomogram validated by Brockman et al. (Cleveland Clinic) was calculated for the present cohort [7]. Actual versus predicted PCSM in our group was 3.1% vs. 3.8% (*p* = 0.640) at 5 years, 9.5% vs. 8.6% (*p* = 0.784) at 10 years, and 16.5% vs. 13.7% (*p* = 0.110) at 15 years. Despite more conservative management without ADT and/or RT in one-third of patients in this cohort, the risk of PCSM did not increase. Another limitation of the present study is the use of retrospective analysis. However, this is conceptually similar to a phase 1 trial in that we explored the safety of no treatment. As such, this analysis was not amenable to a randomized study design, and the retrospective nature allowed for data collection to proceed without bias or intention to compare active observation to systemic treatments. Furthermore, follow-up was robust; 65.6% of all patients received follow-up within the last 5 years, and 71.4% of patients over the age of 80 years and 77.9% of patients with Gleason grade 8–10 received follow-up within the last 3 years. 

## 5. Conclusions

The present study is the first to validate the use of both initial PSADT and continuous PSADT pattern in post-RP management of patients with BCR. Predictive modeling suggests that one-third of patients are at low risk of post-RP recurrence and can be safely managed with active observation with very little risk of PCSM. Furthermore, ad hoc analysis of PCSS suggests PSADT pattern to be a strong predictor of survival outcomes, further emphasizing the need for longitudinal follow-up and continued observation of PC patients following RP. Overall, the initial PSADT and PSADT pattern should be considered for integration into PC management, risk stratification, and decision making when discussing the need for treatment following post-RP BCR. 

## Figures and Tables

**Table 1:** Demographics of BCR patients in the AO (N = 136) and treatment (*n* = 271) groups.

Treatment	No Trmt	Trmt	Total	
	**Count (%)**	**Count (%)**	**Count (%)**	
N, all patients	136 (33.4%)	271 (66.6%)	407 (100%)	
	**Mean (SD)**	**Mean (SD)**	**Mean (SD)**	***p* value**
Age, years	63.5 (7.3)	63.8 (7.2)	63.7 (7.3)	0.677
Adj pre-PSA, ng/mL	8.4 (5.7)	12.6 (16.9)	11.2 (14.3)	0.005
SHIM	19.8 (7.1)	17.9 (7.5)	18.6 (7.4)	0.023
EBL	102.2 (48.4)	96.2 (37.7)	98.2 (41.7)	0.171
BMI	27.0 (3.8)	27.3 (3.8)	27.2 (3.8)	0.467
Prostate weight	51.4 (21.3)	53.5 (19.4)	52.8 (20.1)	0.337
Follow-up, years	7.5 (4.0)	7.7 (4.4)	7.6 (4.3)	0.688
Time to death, years	6.9 (2.7)	7.8 (4.0)	7.6 (3.8)	0.426
Time to earliest treatment	NA	3.0 (7.7)	3.0 (7.7)	
Current PSADT, months	26.0 (19.9)	8.5 (9.1)	15.6 (16.9)	<0.001
PSADT after 0.2, months	39.4 (294.9)	12.6 (48.4)	23.6 (192.6)	0.272
	**Count (%)**	**Count (%)**	**Count (%)**	***p* value**
Margins	36 (26.5%)	109 (40.2%)	145 (35.6%)	0.006
p-stage				<0.001
pT2	67 (49.3%)	70 (25.8%)	137 (33.7%)	
pT3/T4	69 (50.7%)	201 (74.2%)	270 (66.3%)	
Gleason grade group (GGG)				<0.001
1	17 (12.5%)	4 (1.5%)	21 (5.2%)	
2	48 (35.3%)	52 (19.2%)	100 (24.6%)	
3	43 (31.6%)	79 (29.2%)	122 (30.0%)	
4	17 (12.5%)	22 (8.1%)	39 (9.6%)	
5	11 (8.1%)	114 (42.1%)	125 (30.7%)	
PSADT > 0.2 group, months				<0.001
>12	90 (73.8%)	37 (22.6%)	127 (44.4%)	
6 to 12	19 (15.6%)	48 (29.3%)	67 (23.4%)	
<6	13 (10.7%)	79 (48.2%)	92 (32.2%)	
NA	14 *	107 **	121	
DT pattern				<0.001
Increasing	96 (72.7%)	49 (32.7%)	142 (50.7%)	
Decreasing	36 (27.3%)	101 (67.3%)	138 (49.3%)	
NA	4 ***	121 **	127	
PCSM	0 (0.0%)	29 (10.7%)	29 (7.1%)	<0.001
Dead	13 (9.6%)	50 (18.5%)	63 (15.5%)	0.019

* Not enough PSAs prior to non-cancer specific death (*n* = 2), not enough PSAs post-BCR to establish PSA (*n* = 12). ** No PSADT as treatment was initiated based on very rapid PSA progression. *** Not enough PSAs prior to non-cancer specific death (*n* = 2), lost to follow-up (*n* = 1), and after BCR (*n* = 1).

**Table 2:** Full univariate (A) and multivariate (B) regression analysis in all BCR patients (*n* = 407) for no treatment and for initial PSADT (C) and DT pattern (D) models alone.

Outcome: No Treatment			
**A. Univariate Model**	References	Estimated OR (95% CI)	*p*-value
PSADT binary	>12 months vs. <12 months [ref]	8.79 (4.92, 15.71)	<0.001
DT pattern	Increasing vs. decreasing [ref]	6.08 (3.48, 10.62)	<0.001
GGG	4–5 vs. 1–3 [ref]	0.29 (0.17, 0.52)	0.04
Preoperative PSA (continuous)		0.95 (0.91, 0.99)	0.204
P-stage	pT3/4 vs. pT2 [ref]	0.63 (0.38, 1.05)	0.639
Age (continuous)		0.9873 (0.9518, 1.0242)	0.985
**B. Full Multivariate Final Model**		Estimated OR (95% CI)	*p*-value
PSADT binary	>12 months vs. <12 months [ref]	8.93 (4.53, 17.6)	<0.001
DT Pattern	Increasing vs. decreasing [ref]	5.49 (2.81, 10.71)	<0.001
**C. PSADT Binary-Only Model**		Estimated OR (95% CI)	*p*-value
PSADT binary	>12 months vs. <12 months [ref]	8.74 (5, 15.28)	<0.001
**D. DT Pattern-Only Model**		Estimated OR (95% CI)	*p*-value
DT pattern	Increasing vs. decreasing [ref]	5 (2.95, 8.48)	<0.001
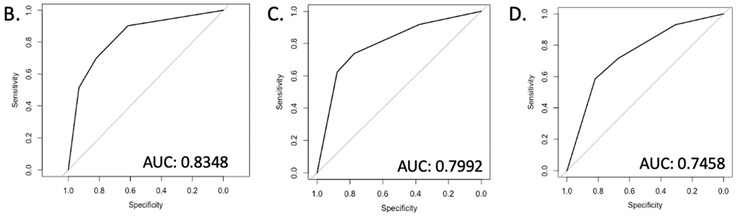			

## Data Availability

The data presented in this study are available on request from the corresponding author.

## Supplementary Materials

The following supporting information can be downloaded at: https://www.mdpi.com/article/10.3390/cancers14174087/s1, Table S1: Univariate and multivariate regression analysis in BCR patients for NO PCSM.
