# Supplementary material for: Prostate-Specific Antigen Doubling Time Kinetics following Radical Prostatectomy to Guide Need for Treatment Intervention: Validation of Low-Risk Recurrences
Source: Cancers (Basel). 2022 Aug 24;14(17):4087. doi: 10.3390/cancers14174087 (PMC9454714; doi:10.3390/cancers14174087)
Supplement: Supplementary file 1 [file cancers-14-04087-s001.zip › cancers-1858615-supplementary.pdf]

Supplementary Table S1: Univariate and multivariate regression analysis in BCR patients for NO PCSM.

Outcome: PCSS		Univariate Model		Multivariate Model 1		Multivariate Model 2	
**Variable**	References	Estimate OR (95% CI)	p-value	Estimate OR (95% CI)	p-value	Estimate OR (95% CI)	p-value
PSADT binary	>12mos vs <12mos [ref]	2.7 (0.55,13.3)	0.565	1.68 (0.32,8.75)	0.526		
DT Pattern	Increasing vs Decreasing [ref]	8.08 (0.99,65.72)	0.08	7.18(0.84,61.59)	0.028	9.72 (1.21,78.09)	0.006
GGG	4-5 vs 1-3 [ref]	0.96 (0.23,3.97)	0.59				
Preoperative PSA (continuous)		0.95 (0.88,1.03)	0.65				
P-stage	pT3/4 vs pT2 [ref]	0.37 (0.08,1.83)	0.259	0.4 (0.08,2.01	0.232		
Age (continuous)		1.06 (0.96,1.17)	0.244	1.07 (0.96,1.18)	0.0.202	1.08 (0.99,1.18)	0.092
